# Linking thyroid dysfunction with chronic kidney disease among Indian patients

**DOI:** 10.6026/973206300212966

**Published:** 2025-09-30

**Authors:** Gayathri Chelamkuri, Nalugotla Lakshmanna, Vadlamudi Chandramohan, Veluri Ganesh

**Affiliations:** 1 Department of Biochemistry, Kurnool Medical College, Kurnool, Andhra Pradesh, India; 2 Department of Biochemistry, Government Medical College, Paderu, Andhra Pradesh, India; 3 Department of Biochemistry, PES University Institute of Medical Sciences and Research, Electronic City, Bengaluru, Karnataka, India

**Keywords:** Chronic kidney disease, hypothyroidism and estimated GFR (eGFR)

## Abstract

The implications of chronic kidney disease are extensive and affect the pituitary-thyroid axis and peripheral metabolism of thyroid hormones causing significant thyroid dysfunction. The present cross-sectional study includes 90 chronic kidney disease diagnosed patients. We investigated for blood parameters like urea, creatinine and thyroid biomarkers. The overall prevalence of thyroid abnormalities in the study group was 65.56%, 59 out of 90 subjects were observed to have derangements in thyroid biomarkers. The Pearson's correlation reveled there was a significant negative correlation between the serum thyroid stimulating hormone levels and the estimated glomerular filtration (r=-0.04, P=0.001**). This study emphasizes the thyroid derangements of clinical relevance in chronic kidney disease patients which may be helpful to clinicians for better patient care.

## Background:

Chronic kidney disease (CKD) encompasses a spectrum of different pathophysiologic processes associated with abnormal kidney function and a progressive decline in glomerular filtration rate (GFR). The CKD is characterized by a sustained estimated glomerular filtration rate eGFR of < 60mL / min / 1.73 m2 for more than three months irrespective of underlying cause [1]. CKD has a significant impact on global health as direct cause of morbidity and mortality and is an important risk factor for cardiovascular disease. Due in part to the rise in risk factors, such as obesity and diabetes mellitus, the number of patients affected by CKD has also been increasing, affecting an estimate of 843.6 million individuals worldwide in 2017 [2]. The overall prevalence of CKD in India is estimated to be around 13.24% according to a recent systematic review and this review indicated a rising trend of CKD from 11.12% during the period 2011 to 2017 to 16.38% between 2018 to 2023 [3]. Kidneys play an important role in the regulation of metabolism and elimination of thyroid hormones. Thyroid gland disorders such as hypothyroidism and euthyroid sick syndrome (ESS) occur very often in stage 5 CKD. Studies also demonstrated the clinical and sub-clinical hypothyroidism states to be independent risk factors for cardiovascular death and all-cause mortality which could be the effects of worsening atherosclerosis in coronary and peripheral vessels and hyperlipidaemia. One of the most important links between thyroid disorders and CKD is uraemia. TSH levels are usually normal with an altered circadian rhythm. In uraemia, the pituitary receptor response to TRH is blunted causing a decrease in TSH release. The response of TSH to TRH is delayed because of the decreased clearance and the increase of half-life of TSH. Abnormal serum constituents found in uremic conditions can also displace T3 and T4 from normal protein binding sites [4, 5-6]. Kidneys typically contributes to iodide clearance primarily by glomerular filtration, this function is impaired in CKD causing an initial increase in thyroidal iodide pool as well as subsequent accumulation of plasma inorganic iodine. According to Wolff-Chaikoff effect, thyroid hormone production could be potentially blocked by increase in total body inorganic iodide. This may explain the slightly higher frequency of goitre and hypothyroidism in patients with CKD [7]. The earliest thyroid dysfunction seen among CKD patients is low T3 level which reflects reduced conversion from T4. The cause is multifactorial ranging from acid base derangements in CKD to the effect of inflammatory cytokines etc. Studies indicated that there is no increased prevalence of hyperthyroidism in CKD. However, hyperthyroidism may accelerate the disease progression in CKD [8]. Mechanisms underlying include: a) increased renal blood flow and increased intraglomerular hypertension leading to increased filtration pressure and consequent proteinuria and direct renal injury; increased mitochondrial energy metabolism and increased free radical generation causing renal injury and oxidative stress also contributes to hypertension [9- 10]. Therefore, it is of interest to evaluate the thyroid interactions in patients with chronic kidney disease.

## Materials and Methods:

This is an cross-sectional study was conducted in the department of Nephrology and Clinical Biochemistry at Government General Hospital, Kurnool from May 2024 to April 2025. A total of 90 chronic kidney disease patients were, sub grouped as shown in Figure 1 (see PDF). Additionally, we recruited 30 ages and gender matched healthy controls. All the study participants were recruited after obtaining approval from institutional ethics committee, and informed consent from all the study participants.

## Criteria of the study:

## Inclusion criteria:

The chronic kidney disease patients diagnosed as per kidney disease improvement global outcomes criteria (KDIGO). The estimated glomerular filtration rate eGFR >90 ml/min considered CKD stage 1 (n=30, Group 2), eGFR 60-79 ml/min considered CKD stage 2 (n=30, Group 3), and eGFR 44-59 ml/min considered CKD stage 3 (n=30, Group 4). The healthy controls without any illness (n=30, Group 3).

## Exclusion criteria:

The patients with known thyroid disorders, diabetes mellitus, metabolic syndrome, acute kidney injury, acute systemic illnesses and pregnant women were excluded from the study.

## Sample collection and methods & Instrumentation:

About 5mL of blood is collected under aseptic conditions into clot activator tubes and the serum obtained is processed. The T3, T4, TSH, FT3, FT4 were assayed as per the standard guidelines by chemi luminescence immunoassay (CLIA) on Beckman Coulter Access 2 analyzer. The other investigations - blood urea and serum creatinine were assayed on Beckman Coulter fully automated chemistry analyzer AU-480 using Urease GLDH method and Modified Jaffe's kinetic method respectively as per the standard guidelines. The eGFR is calculated using Modification of Diet in Kidney Disease (MDRD) application-based formula.

## Statistical analysis:

Data is analyzed using SPSS software. Continuous variables are expressed as mean ± standard deviation and categorical variables are expressed as weighted percentages (%). Pearson's correlation is used to find the correlation between eGFR and serum TSH levels.

## Results:

Table 1 (see PDF) illustrates the baseline characteristics of study variables. The age, total triiodothyronine and free triiodothyronine not shown significant between the CKD patients and controls (P=0.34, 0.52 & 0.46). the urea, creatinine, total thyroxine, free thyroxine, and thyroid stimulating hormone significantly elevated in patients with CKD when compared to controls (P=0.001**). The eGFR was significantly decreased in CKD patients when compared to controls (0.001**). Table 2 (see PDF) shows the comparison of study variables between study subjects. The total triiodothyronine and free triiodothyronine not shown significant between the CKD patients and controls (P=0.98 & 0.19). The urea, creatinine, total thyroxine, free thyroxine, and thyroid stimulating hormone drastically very high in CKD stage 1, to stage 3 patients when compared to controls (P=0.001**). The CKD stage 2 and stage 3 patients has significantly decreased eGFR levels when compared to CKD stage 1 patients when compared to controls (0.001**). The Pearson's correlation coefficient between the serum TSH levels and serum urea, creatinine, eGFR in the study group shown in (Table 3 - see PDF). There were significant positive correlation between TSH and serum urea, creatinine and negative correlation between TSH and eGFR respectively P value is 0. 001*.

## Discussion:

Thyroid hormones (TH) have an important role in kidney growth, development, and homeostasis. Conversely, alterations in the thyroid can affect the glomerular filtration rate (GFR), renal blood flow, tubular function, electrolyte and water balance, and kidney shape and function, all of which lead to changed kidney function [4]. The kidney primarily uses glomerular filtration to aid in the removal of iodine. Patients with CKD who have lower GFR have lower iodine clearance. Iodide absorption in thyroidal tissue and plasma iodide concentration both raise as a result of this. A rise in total body inorganic iodide inhibits the pituitary-thyroid axis, which prevents the generation of thyroid hormones [8]. The higher incidence of hypothyroidism in CKD patients may be explained by these alterations. In the current study, the thyroid hormone status of 90 CKD patients was compared to that of 30 healthy controls. The most prevalent thyroid function anomaly in the current investigation, normal TT3 and their mean TT3 was not significantly different from that of the healthy control. While most CKD patients in this study had elevated TT4 levels, the mean TT4 was statistically significantly higher than that of the healthy controls. In the current investigation, the mean TSH was considerably higher in CKD patients than in controls. Similarly, the recent study found that CKD patients had a significant increase in TSH levels but a non-significant T3 and T4 levels. In contrast, to another study outcome when they examined 50 CKD patients between the ages of 20 and 50. They found that, in comparison to controls, there was a highly significant decrease (P < 0.01) in T3 and T4 levels and an increase in TSH levels [11].

Although hypothyroidism, which is typically linked to a decrease in serum total and free T3, is not unusual, the majority of CKD patients on hemodialysis are euthyroid. This decrease is linked to inflammation, systemic acidity, and endothelial damage. Other previous studies discovered that CKD patients had significantly lower T3 and T4 levels while having the same TSH levels as controls [12]. The CKD patients undergoing conservative treatment or routine hemodialysis revealed a significant decrease in TT3 and TT4, but no discernible changes in TSH levels when compared to the control group. The mean TT3, TT4 and TSH levels were also compared between hemodialysis patients and CKD patients receiving conservative treatment. Compared to individuals receiving conservative treatment, hemodialysis-treated CKD patients had significantly lower mean TT3 and TT4 levels and significantly higher mean TSH levels [13-14]. According to previous study findings, patients with ESRD had significantly lower levels of both FT3 and FT4, whereas those with chronic kidney disease had significantly higher levels of thyroid-stimulating hormone [15, 16- 17]. When another cross sectional study examined fifty CKD patients between the ages of 20 and 50, they discovered comparable outcomes. The patients' T3 and T4 levels were significantly lower and their TSH levels were higher than those of the controls [18]. It is unclear what causes the increased prevalence of hypothyroidism in end-stage renal illness. Nonetheless, it is thought that the accumulation of other harmful compounds in the body, which affect both the central and peripheral systems, and the building of inorganic iodide brought on by decreased kidney excretion may be factors in this phenomenon. Another recent study reported that the fall in estimated GFR was linked with a significant increase in TSH and a noteworthy decrease in FT3 and FT4 [19]. The present study also found significantly increased levels of FT4 levels in CKD patients when compared to controls. The TSH levels positively correlated with urea, creatinine and negatively correlated with estimated eGFR.

## Conclusion:

A loss in renal function will be accompanied by thyroid hormone abnormalities. Treatment strategy planning may benefit from early detection of thyroid disease and its complex interaction with kidney function. In addition to treating renal abnormalities in CKD patients.

## Limitations of the study:

The study group is small and comprised mainly CKD stage 4 and 5. The study is an observational analysis and confined to a single center.
